# Systemic profile of immune factors in an elderly Italian population affected by chronic strongyloidiasis

**DOI:** 10.1186/s13071-020-04391-w

**Published:** 2020-10-15

**Authors:** Natalia Tiberti, Dora Buonfrate, Carmine Carbone, Geny Piro, Zeno Bisoffi, Chiara Piubelli

**Affiliations:** 1 Department of Infectious-Tropical Diseases and Microbiology, IRCCS Sacro Cuore Don Calabria Hospital, Negrar, Italy; 2 Department of Medical and Surgical Sciences, Fondazione Policlinico Universitario A. Gemelli IRCCS, Rome, Italy; 3 Department of Diagnostics and Public Health, University of Verona, Verona, Italy

**Keywords:** *Strongyloides stercoralis*, Chronic strongyloidiasis, Cytokines, Chemokines, Growth factors, Immune response

## Abstract

**Background:**

Strongyloidiasis caused by *Strongyloides stercoralis* is a soil-transmitted helminthiasis affecting an estimated 370 million people and considered one of the most neglected tropical diseases. Although mostly distributed in tropical and subtropical areas, autochthonous infections have also been documented in north-eastern Italy, even though the transmission presumably stopped decades ago. Because of its peculiar auto-infective cycle, strongyloidiasis can persist lifelong, but the pathophysiological mechanisms associated with the maintenance of such a chronic infection are yet to be fully deciphered.

**Methods:**

Serum levels of 23 immune factors were retrospectively assessed in a subgroup of participants in a randomised clinical trial for the treatment of strongyloidiasis (Strong Treat). Here we included Italian subjects born between 1931 and 1964 and diagnosed with strongyloidiasis between 2013 and 2017 (Ss^+^, *n* = 32). Serum samples obtained before (BT) and 6 months (6M AT) after ivermectin treatment, as well as from age- and gender-matched uninfected controls (CTRL, *n* = 34) were analysed.

**Results:**

The assessed immune factors showed a general reduced concertation in Ss^+^ patients and a lack of association with eosinophilia. In our cohort, we did not observe the classical shift towards a type 2 immune response, since Th1 and Th2 cytokines were mostly unaltered. Instead, we observed chemokines as particularly affected by the presence of the parasite, since IL-8, CCL3, CCL4 and CCL5 were significantly reduced in concentration in Ss^+^ subjects compared to CTRL, suggesting that immune cell recruitment to the infection site might be dampened in these patients. This observation was further sustained by a significant increase of CCL4, CCL5 and CCL11 concentrations 6M AT. A significant raised systemic concentration of three growth factors, bFGF, PDGF-BB and IL-7 (haematopoietic growth factor) was also observed post-treatment, indicating a potential involvement in restoring tissue integrity and homeostasis following parasite elimination.

**Conclusions:**

These preliminary data suggest that, in order to survive for such a long period, *S. stercoralis* might suppress host responses that could otherwise result in its ejection. Our results offer novel insights in the potential mechanisms of disease tolerance that might take place during this chronic infection, including a potential T-cell hypo-responsiveness and a role for chemokines.
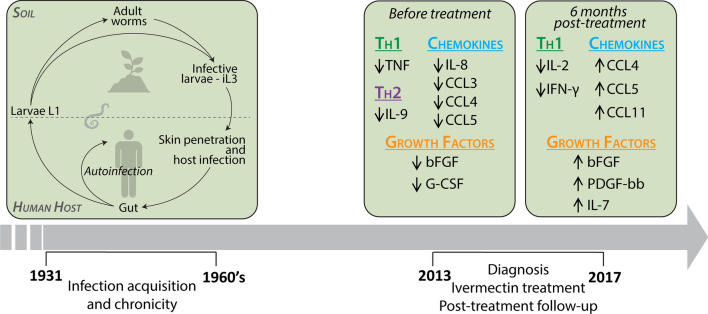

## Background

Strongyloidiasis is a chronic helminthiasis due to *Strongyloides stercoralis* and considered to affect 30–100 million people worldwide [1], although these estimates are regarded as inaccurate and a prevalence of 370 million cases was recently proposed [2]. Strongyloidiasis is primarily endemic in tropical and subtropical regions; however, autochthonous transmission has also been reported in temperate climate areas, including Europe [3]. In Italy, according to a large epidemiological study in six northern provinces, about 8% of Italians born before 1952 and presenting eosinophilia had *S. stercoralis* infection [4], even though the transmission presumably stopped decades ago. The infection caused by *S. stercoralis* can, in fact, last lifelong due to the auto-infective life-cycle, which is peculiar of this nematode [5]. The free-living stage found in soil generates infective iL3 larvae that can penetrate the intact skin of the human host. Once settled in the host’s small intestine, females reproduce by parthenogenesis, producing eggs that hatch already in the gut, so that L1 larvae are released with faeces. Some larvae may mature inside the intestinal lumen into infective larvae that can then penetrate the perianal skin again and complete an autoinfection cycle. This autoinfection allows *S. stercoralis* to complete its life-cycle within the human host perpetuating the infection potentially indefinitely in the absence of further exposure to contaminated soil [5, 6].

Strongyloidiasis is often asymptomatic or presents with non-specific symptoms usually involving the skin, the lung or the gut [6]. Nonetheless, in some immunocompromised subjects, particularly in those receiving corticosteroid therapy or co-infected with human T cell lymphotropic virus type 1 (HTLV-1), strongyloidiasis can be fatal due to the development of hyper-infection or disseminated disease characterised by an accelerated autoinfection responsible for a rapid increase in the parasitic load and the possible dissemination of larvae throughout the host [5, 6].

The lack of a diagnostic gold standard makes the diagnosis of strongyloidiasis cumbersome, especially in low-resource settings, and contributes to the underestimation of the disease prevalence. The traditional microscopic detection of larvae in faeces has unsatisfactory sensitivity; similarly, molecular detection of *S. stercoralis* DNA in stools by PCR is still considered inadequate for screening purposes [7]. Higher sensitivity can be achieved using serological methods, although there are some concerns about the specificity, particularly when used in endemic areas, due to possible cross-reactions with other nematodes [8, 9].

From a pathophysiological point of view, the maintenance of the chronic infection without the development of severe strongyloidiasis seems to be the result of a fine interplay between the host immune system and the pathogen, although the specific mechanisms are yet to be fully deciphered [10]. Indeed, compared to other parasites, little is known about *S. stercoralis* mechanisms of interaction with its human host. Previous clinical and experimental studies have found that, as for most helminths, *S. stercoralis* elicits a response that involves the activation of eosinophils and neutrophils and an increased release of type 2 cytokines, especially interleukin (IL-) 4, IL-5 and IL-13 [11–18]. Additionally, *S. stercoralis* co-infection in patients suffering from different co-morbidities, namely HTLV-1 or type 2 diabetes mellitus, was shown to modulate the host response towards a down-regulation of the Th1 immune response [13, 19–22]. Moreover, conditions that have a major impact on the host immune system, such as co-infection with HTLV-1 or treatment with immunosuppressive drugs, have been postulated to act as “triggers” for the dissemination [6, 11, 23].

In recent years, few studies have investigated the immunological status in human strongyloidiasis, and associations between anti-inflammatory and type 2 cytokines at both the systemic and cellular level were reported in asymptomatic cases. Specifically, subjects suffering from strongyloidiasis were reported to exhibit increased anti-inflammatory and decreased pro-inflammatory cytokines and this profile was reverted after anti-helminthic treatment [14]. Similarly, increased frequencies of CD4^+^ T cells expressing type 2 cytokines (namely, IL-4, IL-5 and IL-13) and decreased frequencies of CD4^+^ T cells expressing type 1 or 17 cytokines were observed in *in vitro* cultures of whole blood in response to *Strongyloides* antigens [13].

In the study here presented, we hypothesised that the retrospective investigation of serum levels of immune factors in a unique population of elderly Italian subjects diagnosed with non-disseminated strongyloidiasis between 2013 and 2017 and supposedly infected for decades, might contribute in further understanding the mechanisms of maintenance of parasitism in chronic strongyloidiasis.

## Methods

### Study population and sample collection

Patients with strongyloidiasis were enrolled in the context of a randomised controlled clinical trial (Strong Treat clinical trial), the objective of which was to evaluate two different ivermectin regimens for the treatment of non-disseminated strongyloidiasis (ClinicalTrial.gov, NCT01570504) [24]. All patients here included were enrolled at the IRCCS Sacro Cuore Don Calabria Hospital (Negrar, Italy) and were subjected to a one-year follow-up with visits and blood examinations performed 6 and 12 months post-treatment.

For the present study, the following inclusion criteria were applied: (i) diagnosis of strongyloidiasis established by detection of *S. stercoralis* larvae in stools and/or positive serology (inclusion of participants in the Strong Treat trial followed a serological threshold criterion, as described in the following paragraph); (ii) Italian origin without any history of travelling to strongyloidiasis endemic areas; (iii) availability of serum samples obtained at baseline (i.e. before treatment administration) and 6 months after treatment. Patients with other known parasitic infections were excluded (Additional file 1: Figure S1).

Age and gender matched non-infected controls were selected among subjects of Italian origin, having a negative serology for strongyloidiasis and whose serum had been stored in our biobank (Tropica Biobank, Protocol n. 50950/2019, approved by the Ethical Committee for Clinical Research of Verona and Rovigo Provinces). As for patients of the Strong Treat clinical trial, subjects with known primary and secondary immunodeficiencies (such as oncological, rheumatological conditions, but also genetic conditions) and those under treatment with steroids, monoclonal antibodies and other immunosuppressant drugs were excluded from the selection.

All sera analysed were collected between 2013 and 2017, aliquoted and stored at − 80 °C until further use.

### Diagnosis of strongyloidiasis and response to treatment

Diagnosis of strongyloidiasis was established as reported by Buonfrate et al*.* [24]. Briefly, presence of larvae in stools was evaluated through direct parasitological examination by microscopy and/or agar plate culture. The presence of anti-*Strongyloides* antibodies in serum was assessed using either an in-house immunofluorescent test (IFAT) [25] or commercially available ELISA assays (*Strongyloides* serum ELISA, IVD Research, Carlsbad, CA, USA, or *Strongyloides ratti* ELISA, Bordier Affinity Products, Crissier, Switzerland). According to the Strong Treat clinical trial, the diagnosis of strongyloidiasis was established based either on the detection of larvae in stools or on a positive serology at high titre, i.e. IFAT titre of at least 1:160, IVD Research ELISA normalised optical density (OD) ≥ 2, Bordier ELISA normalised OD ≥ 2.5 [24].

The primary outcome was defined as clearance from infection (established as negative agar plate culture and negative serology, or a positive serology with a two-fold decrease in IFAT titre or ELISA normalised OD compared to baseline) assessed 12 months after treatment. The clearance of infection 6 months after treatment (defined as per primary outcome) was defined among secondary outcomes.

Non-infected controls were defined as having a negative IFAT result (i.e. IFAT titre = 0).

### Multiplex bead suspension assay (Bio-Plex)

The serum concentration of 27 cytokines, chemokines and growth factors were simultaneously quantified using the Bio-Plex Pro^TM^ Human Cytokine 27-plex immunoassay (Bio-Rad, Hercules, CA, USA) on a Bio-Plex 200 System (Bio-Rad). The concentration of the following targets was assessed: IL-1β; IL-1ra; IL-2; IL-4; IL-5; IL-6; IL-7; IL-8 (or C-X-C motif chemokine—CXCL8); IL-9; IL-10; IL-12p70; IL-13; IL-15; IL-17A; eotaxin (C-C motif chemokine 11—CCL11); basic fibroblast growth factor (bFGF); granulocyte-colony stimulating factor (G-CSF); granulocyte-macrophage colony-stimulating factor (GM-CSF); interferon gamma (IFN-γ); interferon gamma-induced protein 10 (IP-10, also known as CXCL10); monocyte chemoattractant protein 1 (MCP1 or CCL2); macrophage inflammatory proteins 1α (MIP-1α or CCL3); macrophage inflammatory proteins 1β (MIP-1β or CCL4); platelet-derived growth factor subunit B (PDGF-BB); RANTES (CCL5); tumour necrosis factor (TNF); and vascular endothelial growth factor (VEGF).

Samples were randomly distributed across two 96-well plates and assessed according to the manufacturer’s instructions, using 12.5 µl of serum for each sample. Quality controls consisting of a pool of sera spiked with three different known amounts of standard (i.e. zero, medium or high) were tested in duplicate on each plate. The performance of the assay, assessed for each target individually, was evaluated through the percentage recovered concentration (comprised in the range 75–125%) and the percentage coefficient of variation on replicates (CV below 20%) measured for each standard within a plate. The variability between plates, was assessed through the % CV on internal quality controls (geometric mean on the CV of 6%). In order to avoid missing values, arbitrary values corresponding to the mean lowest concentration observed on the two plates divided by two or to the mean highest observed concentration multiplied by two was assigned to all samples out of range (OOR).

Considering all the above-mentioned criteria, IL-5, IL-10, IL-15 and GM-CSF were excluded from further analyses due to the high variability between plates or very high frequency (≥ 89%) of OOR values, which hampered a proper calculation of their concentration.

### Statistical analysis

Statistical analyses were performed using STATA 14.0 (StataCorp LLC, College Station, TX, USA) and GraphPad Prism v 8.4.0 (GraphPad Software, San Diego, CA, USA). Comparisons between *Strongyloides-*infected patients and uninfected controls were performed using the Mann-Whitney U-test, while pre- and post-treatment paired comparisons were assessed with the Wilcoxon signed rank test. All tests were two-tailed and the significance level was set at 0.05.

Non-parametric Spearman correlation and univariate regression analyses were performed to assess the association and the dependence between absolute eosinophil count measured on admission and cytokine concentrations.

Receiver operator characteristic (ROC) curves were built to assess the ability of selected variables in discriminating between *Strongyloides-*positive patients (Ss^+^) and uninfected controls. For each variable, the best cut-off was defined through the Youdenʼs index computing the best combination of sensitivity (SE) and specificity (SP). Marker combination was evaluated using PanelomiX [26] considering all immune factors significantly altered in Ss^+^ patients at baseline, eosinophilia and white blood cell (WBC) count as variables. Only panels comprising a maximum of three markers were allowed and optimisation of the global accuracy was chosen for combination and cut-off selection.

## Results

### Population characteristics

The demographic description of patients suffering from strongyloidiasis (Ss^+^, *n* = 32) at baseline and of uninfected control subjects (CTRL, *n* = 34) is summarised in Table 1. As shown, no differences in age or gender were detected between the two groups, while Ss^+^ subjects showed a significantly higher eosinophil count, expressed as both absolute count (Mann-Whitney U-test: *Z* = − 4.057, *P* < 0.0001) and percentage of total WBC (Mann-Whitney U-test: *Z* = − 2.983, *P* = 0.0029). A slight, yet significant, increased WBC count was also recorded in infected subjects (Mann-Whitney U-test: *Z* = − 2.066, *P* = 0.0388).

**Table 1.** Demographic description of uninfected and *S. stercoralis* infected (baseline) subjects

	CTRL (*n* = 34)	Ss^+^ (*n* = 32)	*P-*value
Gender, F (*n*)	13 (38%)	13 (41%)	ns
Age (years), median (IQR)	72 (61.5–78.0)	74 (63.5–78)	ns
Eosinophils (cells/µl), median (IQR)	290 (100–622.5)	815 (550–1078)	< 0.0001
Eosinophils (%), median (IQR)	3.79 (1.515–7.425)	10.45 (6.4–14.55)	0.0029
WBC (cells/µl), median (IQR)	6305 (5675–12,230)	7470 (6600–9305)	0.0388
Geographical origin, Italy (*n*)	34	32	
*S. stercoralis* stool exam, *n* (%)			
Positive	0	15 (47)	
Negative	16 (47)	16 (50)	
Unknown	18 (53)	1 (3)	
*S. stercoralis* serology, *n* (%)			
Positive	0	32 (100)	
Negative	34 (100)	0	

*Notes*: Statistical differences were assessed with the Mann-Whitney U-test, except for gender, which was tested with Fisher’s exact test. Statistical significance was set at *P* < 0.05

*Abbreviations*: CTRL, uninfected controls; Ss^+^, *S. stercoralis* infected individuals; IQR, interquartile range; WBC, white blood cells; ns, not significant

The clinical and haematological description of Ss^+^ patients at baseline (BT) and 6 months after treatment (6M AT) is compared in Table 2. A significant decreased eosinophil count was observed 6 months after treatment (absolute count, Mann-Whitney U-test, *Z* = 4.451, *P* < 0.0001), while the WBC count remained unvaried. At baseline, 47% of patients had *S. stercoralis* larvae in their stools and 63% had a baseline IFAT titre > 160; this latter proportion significantly decreased to 3% 6 months after treatment. The majority of patients were also tested by qPCR, a method that is recommended as a confirmatory test rather than as a primary screening tool [7]. Indeed, 50% of patients were negative at baseline, although this proportion significantly increased to 88% after treatment.

**Table 2.** Clinical description of infected subjects before (BT) and 6 months after treatment (6M AT)

	Baseline—BT (*n* = 32)	6M AT (*n* = 32)	*P-*value
Eosinophils (cells/µl), median (IQR)	815 (550–1078)	245 (117.5–367.5)	< 0.0001
Eosinophils (%), median (IQR)	10.45 (6.4–14.55)	3.16 (1.465–5.6)	< 0.0001
WBC (cells/µl), median (IQR)	7470 (6600–9305)	7425 (6168–8308)	ns
*S. stercoralis* stool exam, *n* (%)			< 0.0001
Positive	15 (47%)	0	
Negative	16 (50%)	29 (91%)	
Unknown	1 (3%)	3 (9%)	
*S. stercoralis* IFAT titre, *n* (%)^a^			< 0.0001
≤ 160	11 (34%)	28 (88%)	
> 160	20 (63%)	3 (9%)	
*S. stercoralis* qPCR, *n* (%)			< 0.0001
Positive	14 (44%)	1 (3%)	
Negative	16 (50%)	28 (88%)	
Unknown	2 (6%)	3 (9%)	
Clinical symptoms BT, *n* (%)			
Present	22 (69%)	na	
Absent	10 (31%)	na	
Detailed clinical symptoms^b^, *n* (%)			
Pruritus	17 (77%)	na	
Skin rash	9 (41%)	na	
Abdominal pain/distension	3 (14%)	na	
Respiratory symptoms	6 (27%)	na	
Clinical symptoms 6M AT^b^, *n* (%)			
Improved	na	13 (59%)	
Persist	na	7 (32%)	
Ceased	na	2 (9%)	
Response to treatment 12M AT^c^, *n* (%)			
Yes	24 (75%)	na	
No	8 (25%)	na	
Response to treatment 6M AT^d^, *n* (%)			
Yes	22 (69%)	na	
No	10 (31%)	na	

^a^Missing information for one subject, who had positive serology by ELISA

^b^Computed over the 22 subjects with clinical manifestations at baseline

^c^Response determined 12 months after treatment completion as primary outcome

^d^Response determined 6 months after treatment as secondary outcome

*Notes*: Statistical significance was assessed using the Wilcoxon signed rank test for continuous variables or with the Fisher’s exact test for categorical variables. Statistical significance was set at *P* < 0.05

*Abbreviations*: IQR, interquartile range; WBC, white blood cells; na, not applicable; ns, not significant

The majority (69%) of Ss^+^ subjects presented clinical symptoms on admission (defined as pruritus, skin rash, abdominal pain/distension, respiratory distress), 41% of which having two or more manifestations. Six months post-treatment clinical manifestations were improved in 59% of subjects presenting symptoms at baseline, ceased in 9% and persisted in 32%. Overall, among the 32 patients with strongyloidiasis here tested, 69% were considered to have responded to therapy as soon as 6 months post-treatment, and 75% had responded by the end of the 12-month follow-up.

### Infected subjects show decreased systemic levels of immune factors compared to uninfected controls

**Fig. 1.**
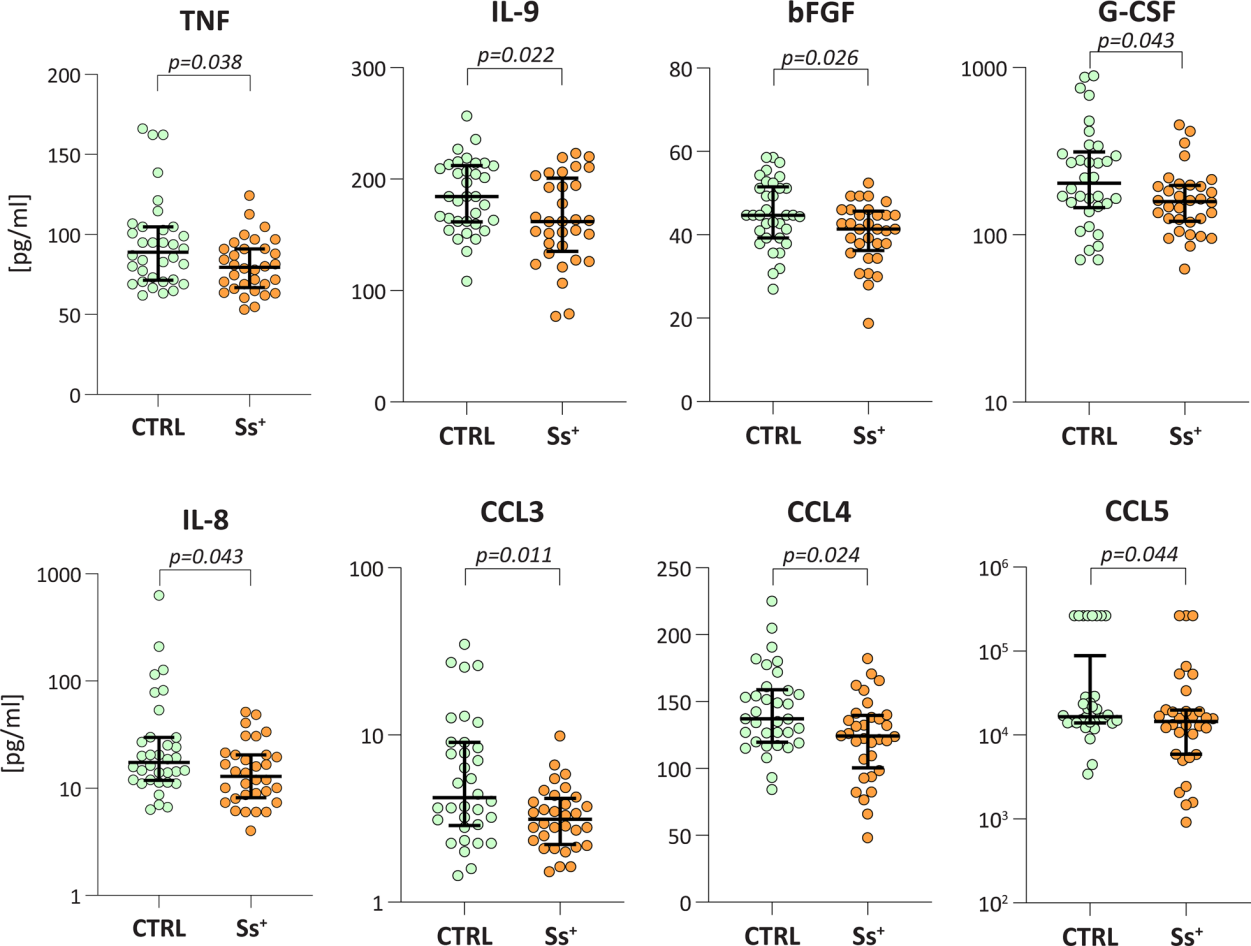
Immune factors significantly altered in infected patients at baseline. Scatter plots showing the decrease in immune factor concentrations in serum from *S. stercoralis*-infected patients (Ss^+^, *n* = 32) compared to uninfected controls (CTRL, *n* = 34). The line on each graph represents the median concentration and bars the interquartile range. Statistical significance was assessed using the Mann-Whitney U-test, and the exact *P*-value is reported on each plot

In the population of elderly Italian subjects here investigated, we observed an overall decreased concentration of immune factors in Ss^+^ compared to CTRL (Fig. 1, Additional file 1: Table S1). Among the Th1 cytokines assessed (i.e. IL-2, IL-12p70, TNF and IFN-γ), only TNF was significantly decreased in Ss^+^ subjects (Mann-Whitney U-test: *Z* = 2.073, *P* = 0.0382). Similarly, IL-9 was the only Th2 cytokine significantly altered (Mann-Whitney U-test: *Z* = 2.284, *P* = 0.0224) among those tested (IL-4, IL-6, IL-9 and IL-13).

In our population, chemokine concentrations were particularly affected by *S. stercoralis* infection. Indeed, four out of seven chemokines, i.e. IL-8 (Mann-Whitney U-test: *Z* = 2.021, *P* = 0.0432), CCL3 (Mann-Whitney U-test: *Z* = 2.560, *P* = 0.0105), CCL4 (Mann-Whitney U-test: *Z* = 2.258, *P* = 0.0239) and CCL5 (Mann-Whitney U-test: *Z* = 2.019, *P* = 0.0435), were significantly diminished in infected subjects compared to CTRL. Similarly, infection was also associated with an altered profile of two growth factors, namely bFGF and G-CSF (Mann-Whitney U-test: *Z* = 2.225, *P* = 0.0261 and *Z* = 2.028, *P* = 0.0426, respectively). Correlations between immune factors and age were computed in order to assess for dependency in our population of elderly individuals. A significant, although weak, correlation was recorded only for IL-2 (Spearman correlation: *rho* = 0.2747, *P* = 0.0256), IL-12p70 (Spearman correlation: *rho* = 0.3137, *P* = 0.0103) and CCL5 (Spearman correlation: *rho* = − 0.3775, *P* = 0.0018) (Additional file 1: Table S2).

ROC analysis was used to further evaluate the ability of Ss^+^-associated immune factors to discriminate between the two groups of subjects. TNF, IL-8, IL-9, CCL3, CCL4, CCL5, bFGF and G-CSF discriminated between Ss^+^ and controls with percent area under the curve (AUC) ranging from 64.4% to 68.3%, with CCL3 showing the best individual accuracy defined as the best combination of SE and SP (96.9% and 38.2%, respectively) (Additional file 1: Table S3a). When assessed in combination with eosinophilia (i.e. > 400 cells/µl) and absolute WBC count, a panel comprising eosinophilia, IL-9 and CCL3 was highlighted as having 87.6% AUC (85.3% SP–87.5% SE), as soon as two out of the three variables are above (or below) their respective cut-offs. This combination significantly improved the discriminatory ability of eosinophilia (*P* = 0.0005—De Long’s test), which alone was the best predictor (Additional file 1: Table S3a, b).

### Ivermectin treatment induces changes in the systemic concentrations of immune factors

**Fig. 2.**
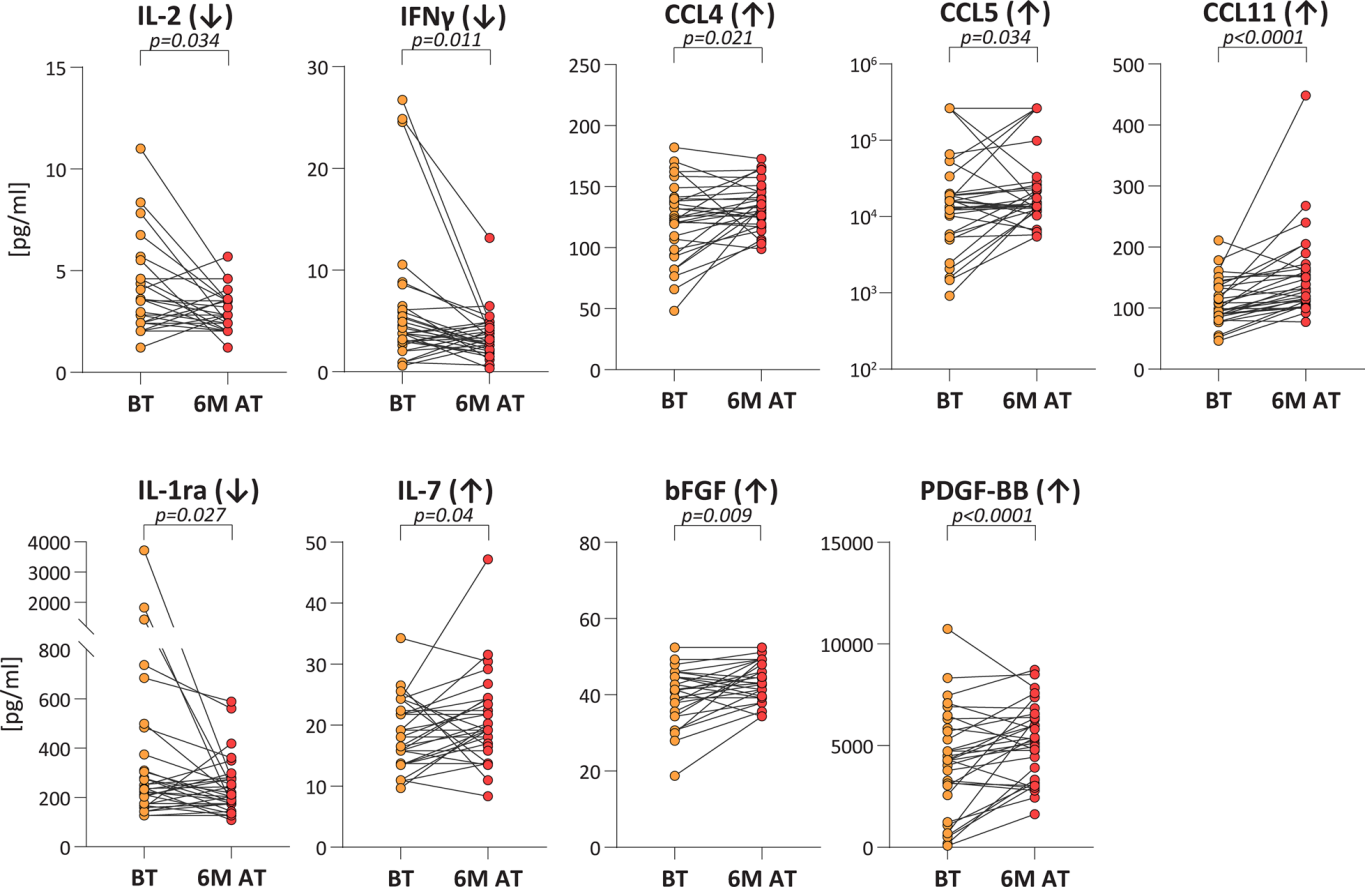
Immune factors significantly altered after ivermectin treatment. Line plots showing the variation in immune factor concentrations observed in serum from *S. stercoralis-*infected patients (Ss^+^, *n* = 32), before treatment (BT) and 6 months after ivermectin treatment (6M AT). The profile for each individual patient is reported. The arrow beside each molecule indicates whether a significant increase or decrease in systemic concentration was observed in Ss^+^ patients. Statistical significance was assessed using the Wilcoxon signed rank test and the exact *P*-value is reported on each plot

When the concentration of immune factors assessed before treatment was compared to the one measured 6 months after treatment, we observed a decrease in two Th1 cytokines and an increase in chemokines and growth factors (Fig. 2, Additional file 1: Table S1). Among Th1 cytokines, the concentrations of IL-2 (Wilcoxon signed rank test: *Z* = 2.124, *P* = 0.0337) and IFN-γ (Wilcoxon signed rank test: *Z* = 2.534, *P* = 0.0113), which were unaltered at baseline compared to controls, dropped following treatment. TNF, which was decreased at baseline compared to controls, remained unaltered.

CCL4 and CCL5 chemokines showed a significantly increased concentration (Wilcoxon signed rank test: *Z* = − 2.309, *P* = 0.0209 and *Z* = − 2.122, *P* = 0.0338, respectively) when measured after treatment, with a reverted profile compared to baseline. Six months after ivermectin treatment, the concentration of CCL11 rose very significantly (Wilcoxon signed rank test: *Z* = − 4.226, *P* < 0.0001) from the same levels as controls observed at baseline. Three growth factors, IL-7 (hematopoietic growth factor) (Wilcoxon signed rank test: *Z* = − 2.051, *P* = 0.0403), bFGF (Wilcoxon signed rank test: *Z* = − 2.625, *P* = 0.0087) and PDGF-BB (Wilcoxon signed rank test: *Z* = − 2.786, *P* = 0.0053) showed raised concentration after treatment as well. Of these, bFGF was also decreased at baseline compared to CTRL. Finally, IL-1ra had a decreased concentration after treatment compared to the baseline (Wilcoxon signed rank test: *Z* = 2.216, *P* = 0.0267). All other targets remained unvaried after treatment.

### Variation in immune factor concentration according to the presence of clinical symptoms in Ss^+^ subjects at baseline

**Fig. 3.**
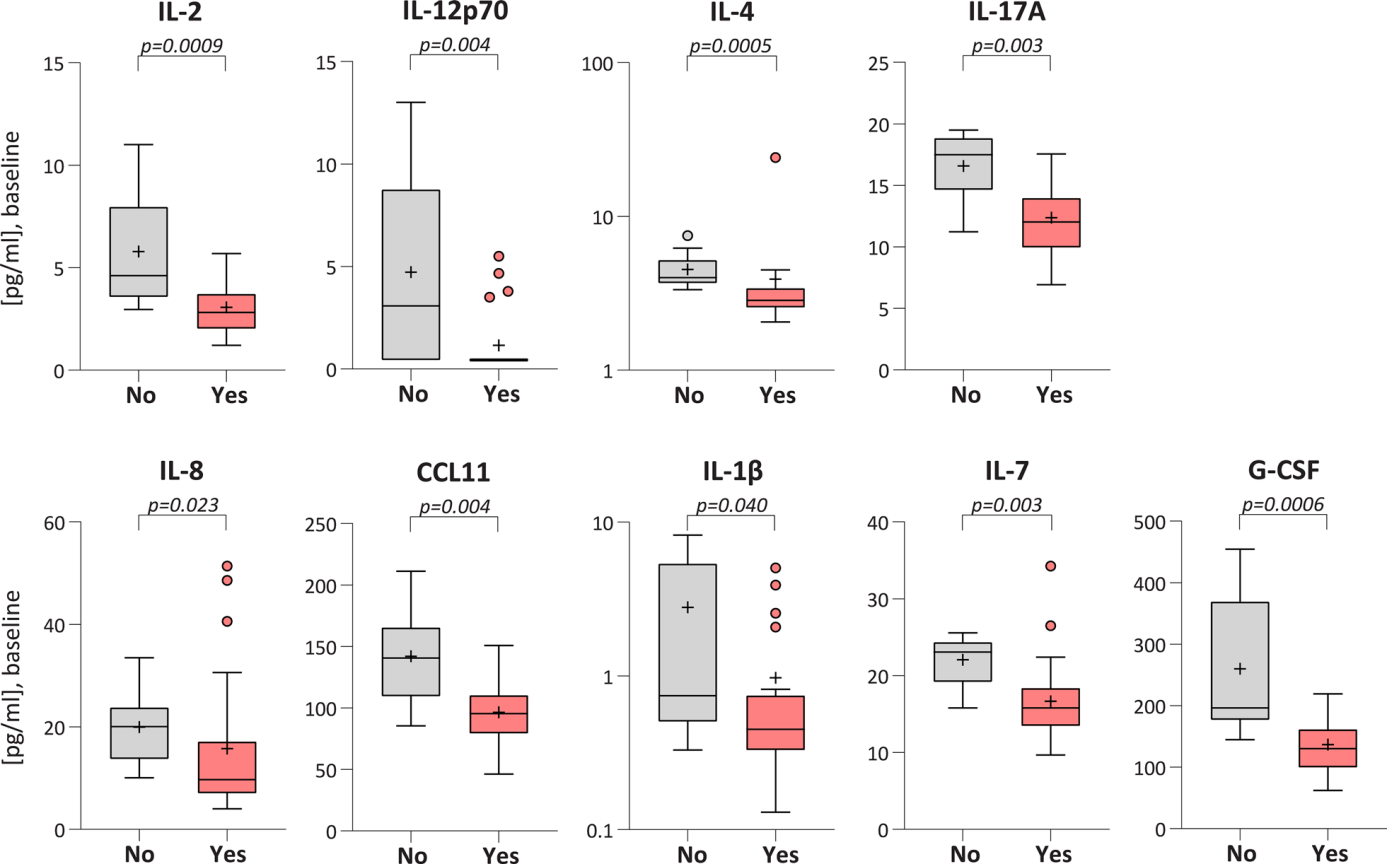
Immune factors significantly altered according to clinical symptoms. Tukey box-plots showing the variation in immune factor concentrations observed in *S. stercoralis*-infected patients classified according to the absence (no, *n* = 10) or the presence (yes, *n* = 22) of clinical symptoms at baseline. The ‘+’ on each plot represents the mean. Statistical significance was assessed using the Mann-Whitney U-test, and the exact *P*-value is reported on each plot

Sixty-nine percent of subjects with strongyloidiasis presented clinical symptoms before treatment (Table 2). In order to evaluate a potential association between the immune status and clinical manifestations, we compared the circulating levels of the 23 studied factors in Ss^+^ patients with (*n* = 22) or without (*n* = 10) clinical manifestations at admission (Fig. 3, Additional file 1: Table S4). Among the tested molecules, 9 showed altered concentration (Mann-Whitney U-test): IL-2 (*Z* = 3.308, *P* = 0.0009); IL-12p70 (*Z* = 2.856, *P* = 0.0043); IL-4 (*Z* = 3.504, *P* = 0.0005); IL-17A (*Z* = 3.004, *P* = 0.0027); IL-8 (*Z* = 2.278, *P* = 0.0227); CCL11 (*Z* = 2.887, *P* = 0.0039); IL-1β (*Z* = 2.057, *P* = 0.0396); IL-7 (*Z* = 2.980, *P* = 0.0029); and G-CSF (*Z* = 3.416, *P* = 0.0006). All these molecules were lower in concentration in patients with symptoms compared to those without symptoms, which instead exhibited higher levels than controls. Considering the targets modulated by *S. stercoralis* infection (Fig. 1), only IL-8 and G-CSF were also affected by the clinical manifestations, suggesting that the results observed for the comparison Ss^+^ *vs* CTRL might be influenced by patients with clinical signs that present a particularly low concentration of these molecules. All other factors significantly decreased in Ss^+^ *vs* CTRL were not associated with clinical manifestations.

### Immune factors associated with strongyloidiasis at baseline are independent from eosinophil count

**Fig. 4.**
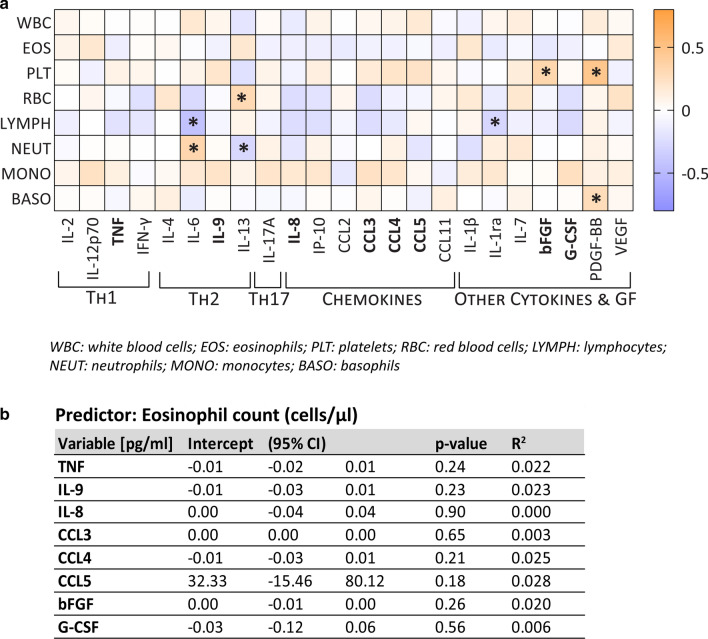
Relation between immune factors and eosinophilia. **a** Correlation between haematological parameters and the serum concentration of immune factors. Correlation with WBC and EOS was computed on the entire population (*n* = 66, Ss^+^ *n* = 32, CTRL *n* = 34), correlation with the other cell types was computed on *n* = 53 patients (Ss^+^ *n* = 19, CTRL *n* = 34) due to missing information for some infected subjects. Significant correlations (*P* < 0.05) according to the Spearman statistics are marked with an asterisk. Colour-code indicates spearman rho coefficient. **b** Univariate regression analysis showing the independence between eosinophil absolute count (cells/µl) and the serum concentration of the immune factors decreased in strongyloidiasis at baseline

Eosinophilia has been long time considered as an indicator of suspicion of *S. stercoralis* infection [27, 28], although its utility for screening is still debated [29]. We thus assessed whether the immune factors highlighted as significantly associated with Ss^+^ at baseline, were also associated with the absolute count of eosinophils. As shown in Fig. 4a, according to the Spearman statistics no correlation was observed between eosinophil count, nor WBC, at admission and any of the assessed molecules. Significant *(P* < 0.05) positive or negative correlations, with Spearman indices varying from low (*rho* = |0.28|) to moderate (*rho* = |0.47|) were instead observed for the following molecules: IL-6 and lymphocytes (*rho* = − 0.4288) or neutrophils (*rho* = 0.3415), IL-13 and RBC (*rho* = 0.3111) or neutrophils (*rho* = − 0.2781), IL-1ra and lymphocytes (*rho* = − 0.2879*)*, bFGF and platelets (*rho* = 0.3310), PDGF-BB and platelets (*rho* = 0.4744) or basophils (*rho* = 0.2817) (Fig. 4a). No correlation was recorded between any haematological parameter and chemokines. The independence between the studied molecules and eosinophilia was also confirmed by linear regression analysis (Fig. 4b) where an absence of association between the assessed variables was observed.

## Discussion

In the present study, we investigated the systemic levels of 23 immune mediators, including cytokines, chemokines and growth factors, in the serum of Italian subjects affected by non-disseminated strongyloidiasis. Indeed, these subjects born between 1931 and 1964 and diagnosed with strongyloidiasis between 2013 and 2017, presumably contracted the infection decades ago, when transmission was still active in Italy as in other Mediterranean countries [4, 30, 31]. Since none of the subjects here investigated had visited endemic countries, they were not exposed to re-infection and did not present co-infection with other soil-transmitted helminths. We believe that this population represents a unique opportunity to study the mechanisms of maintenance of parasitism and of disease tolerance in strongyloidiasis.

As for other helminths, *S. stercoralis* has been shown to modulate the host immune system towards a predominant Th2 response [11, 12, 18, 32–34]. In *in vitro* and *ex vivo* studies analysing samples from patients affected by pathological conditions known to elicit a type 1 response, a shift from a Th1 to a Th2 response was reported when co-infected with *S. stercoralis* [18–20, 22, 35]. According to evidence mainly drawn from murine studies, intestinal epithelial cells (IECs) contribute to elicit type 2 immunity, through the release of alarmins (including IL-25 and IL-33). These cytokines have been reported to stimulate tissue-resident type 2 innate lymphoid cells (ILC2) to release Th2 cytokines (such as IL-4, IL-5, IL-9 and IL-13) involved in the recruitment of eosinophils and alternatively activated macrophages, and in promoting worm expulsion and wound healing [36–39]. In agreement with these observations increased circulating levels of Th2 and anti-inflammatory cytokines were reported in a population of subjects affected by strongyloidiasis from endemic areas [14]. A number of studies investigating directly patients’ plasma or isolated lymphocytes cultured *in vitro*, have further revealed the association between *S. stercoralis* infection and raised type 2 cytokines, while type 1 (especially IFN-γ) and type 17 factors were reduced (Additional file 1: Table S5). Conversely, in our population of long-lasting infections, we did not observe this classical profile but rather a general decrease in immune factors, with chemokines and growth factors being the most affected groups of molecules. Type 1 and type 2 cytokines exhibited, instead, profiles similar to those observed in control subjects.

The co-evolution over millennia of helminths and their human host has likely contributed to the adaptation of the host immune system to tolerate these parasites [38]. *Strongyloides stercoralis* exemplifies this aspect in that, due to its auto-infective cycle, it can generate a chronic infection lasting indefinitely, which can even remain asymptomatic for decades [30]. It has been proposed that disease tolerance might establish during chronic infections as both an alternative mechanism of host defence and as a tool for the parasite to dampen resistance (i.e. worm expulsion) thus ensuring the continuation of its life-cycle [38, 40, 41]. Moreover, it has been proposed that infective and auto-infective larvae might be associated with different host responses [12, 42].

With few exceptions, in our population, overall, we did not observe differences in type 1 and type 2 cytokines between chronically infected patients and uninfected controls, nor after treatment of infected subjects. Although they should be confirmed on a larger number of samples, these results open the question whether mechanisms of T-cell hypo-responsiveness might contribute to the establishment of long-lasting *S. stercoralis* infections. Indeed, a suppressed type 2 immunity, characterised by hypo-responsive Th2 cells with impaired production of Th2 cytokine, has already been associated with chronicity in murine schistosomiasis and filariasis [43–45]. More in depth functional analysis of T-cell responsiveness from chronically infected patients from non-endemic countries - as those here investigated - should thus be carried out to further understand the role of T cells in disease tolerance and in the response to auto-infective larvae in human strongyloidiasis.

As previously mentioned, in our population we observed a number of chemokines to be affected by both the infection and the treatment. The functional role of chemokines in driving immune cell recruitment in strongyloidiasis remains largely unexplored. To the best of our knowledge, the association between circulating chemokines and *S. stercoralis* infection has only been assessed in patients either co-infected with tuberculosis [46] or suffering from type 2 diabetes [21]. Although patients exclusively suffering from strongyloidiasis were not included, both those studies showed chemokines down-modulation in patients with strongyloidiasis and a reverted profile after anti-helminthic treatment. These results partly agree with those observed in the present work, in which, a significant decrease in inflammatory chemokines IL-8, CCL3, CCL4 and CCL5 was observed at baseline in infected patients compared to controls, and a restored concentration was detected after treatment for CCL4 and CCL5; CCL11 also showed a raised concentration AT. CCL3, CCL4, CCL5 and CCL11 partly share the same receptors (i.e. CCR1, CCR3 and CCR5) which are mainly expressed on monocytes and macrophages, basophils, eosinophils and T cells [47]; while IL-8 is mainly implicated in neutrophil trafficking. Being involved in the migration of immune cells to the site of inflammation, these molecules might play an important role in the resistance to the infection, and thus in parasite elimination. A decreased chemokine concentration in chronically infected patients might instead be associated with an impaired recruitment of inflammatory cells (including eosinophils, macrophages, neutrophils and NK cells) to the site of inflammation and, although speculative, it could be hypothesised that this could contribute to disease tolerance.

It is worth noticing that despite the baseline eosinophilia, eosinophil chemo-attractants CCL11 and CCL5 did not correlate with eosinophil count and their concentration was either unaltered (CCL11) or decreased (CCL5) in *S. stercoralis-*infected subjects. Moreover, the significant drop in eosinophil count observed following ivermectin treatment was not accompanied by a decrease in CCL11 and CCL5 concentration, which instead significantly increased. Eosinophil response to treatment in strongyloidiasis might be variable. For instance, in our cohort, as well as in the Strong Treat clinical trial, a significant decrease in eosinophil count was recorded as soon as 17 days post-treatment (data not shown) [24], while Anuradha et al. [14] did not observe differences six months after treatment. Importantly, it has been reported that *S. stercoralis*-derived factors exhibit chemoattractant properties on murine eosinophils *in vitro* upon IL-5 priming, indicating that mechanisms other than classical host chemokines contribute to eosinophil chemotaxis [48]. More in depth functional analyses of eosinophils isolated from these patients should be carried out to more precisely define the role of these cells and of associated cytokines in the pathogenesis of chronic strongyloidiasis. Nonetheless, in our dataset CCL11 exhibited a highly significantly raised systemic concentration following treatment, while at baseline showed the same level as controls. At baseline, this chemokine was also significantly more abundant in the serum of asymptomatic patients compared to those presenting with clinical manifestations. Based on CCL11 investigations in other pathological conditions and its role in eosinophil chemotaxis to the infection site [49, 50], it could be hypothesised that this chemokine could be involved in eosinophil recruitment to contribute to parasite elimination and tissue remodelling following treatment.

Not surprisingly, some growth factors showed altered concentrations in the analysed samples with bFGF, PDGF-BB and IL-7 (or haematopoietic growth factor) being increased post-treatment, suggesting that these molecules might be involved in restoring tissue integrity and homeostasis following parasite elimination. A Th2 mediated repair has, in fact, been hypothesised to occur in helminthiasis and a potential role for wound healing processes in both disease tolerance and resistance has been proposed [51].

Non-disseminated chronic strongyloidiasis can be either asymptomatic or associated with general clinical symptoms [6]. In our population, a number of cytokines were significantly lower in concentration in patients with symptoms. Of these, only IL-8 and G-CSF were associated with infection at baseline compared to controls indicating that, at least for these cytokines, the results could be influenced by the particularly low levels observed in symptomatic patients. Since all other molecules associated with the infection were not influenced by the presence of symptoms, it is likely that their levels reflect an alteration of the immune status as a result of the chronic presence of the parasite. Nonetheless, we cannot exclude that mechanisms other than disease tolerance might establish in these patients and that the overall decrease in immune factors might be exacerbated in patients with a slightly more severe clinical presentation. Indeed, patients’ stratification according to clinical manifestations revealed an overall lower, although not significant, concentration of immune factors in symptomatic patients. Interestingly, the molecules the concentration of which was significantly lower in symptomatic patients, exhibited higher concentrations in asymptomatic patients when compared to controls. Despite the limited number of analysed samples, this result could indicate that a different response might be occurring in this two sub-groups.

In non-endemic countries, increased eosinophil counts might raise, under certain circumstances, suspicion of strongyloidiasis. In the attempt to extend the potential utility of circulating immune mediators beyond disease pathophysiology, we also evaluated their potential for discriminating between infected and uninfected patients. Although none of the molecules in exam showed individually high accuracy (≤ 68%), the combination of IL-9 and CCL3 with eosinophil count, significantly improved the accuracy of the latter in discriminating between the two groups. This result, although preliminary and performed on a limited dataset, highlights the importance of the host-response to the infection not only to understand the mechanisms of disease but also as potential biomarker to highlight individuals possibly at risk of chronic infection, deserving further investigations.

This study presents some limitations that should be taken into account. First, since exploratory, we decided to assess a wide commercial panel of cytokines. Although some relevant novel aspects were revealed, chemokines for instance, some other important mediators such as alarmins (IL-25 and IL-33) and regulatory cytokines as IL-27, IL-37 and TGF-β were not examined. Moreover, some key mediators as IL-5 and IL-10 were not efficiently measured, although included in the experimental panel. In order to evaluate the immune response in a broader context, investigations should also be extended to such molecules.

The study population encompasses elderly subjects that, in addition to strongyloidiasis, might suffer from age-related pathologies or conditions. To minimise potential biases, the subjects of the control group were selected to match patients’ age, so that the unknown presence of potential age-related conditions would be represented among the two groups. Nonetheless, we cannot exclude that such conditions might also influence the level of the assessed immune factors. Only a limited number of patients was available for the present study; in the future, investigations should be extended to a larger number of samples, ideally collected in a multi-centre study. In order to be able to evaluate the trend in the host immune response on a relatively small population and since the exploratory nature of our study, we did not perform a statistical correction for the comparison of the 23 factors on the same population. The extension of the study to a larger population will allow achieving a higher statistical power and obtaining more robust results, with potential clinical implications.

## Conclusions

The immune response to *S. stercoralis*, and to helminths in general, has largely been studied in animal models [52]. Although useful to evaluate the response in a controlled system, these models can mimic only some of the aspects of the human infection. We believe that the here analysed population offers a unique window to study the host response to *S. stercoralis* auto-infection in chronic long lasting human strongyloidiasis in the absence of re-infection. Our preliminary results revealed novel insights in the potential mechanisms of disease tolerance that might take place during this chronic infection, including a potential T-cell hypo-responsiveness and a role for chemokines. The peculiar auto-infective cycle of *S. stercoralis* makes this parasite unique among other helminths and this might explain the difficult generalisation of some known pathophysiological aspects to *S. stercoralis*. More in depth investigations on clinical samples from chronically infected patients not subjected to re-infection will contribute in elucidating functional aspects of the maintenance of parasitism in strongyloidiasis. Moreover, understanding such mechanisms will also pave the way for studies on the association between chronic strongyloidiasis and susceptibility to autoimmune diseases.

## Supplementary information

**Additional file 1: Figure S1.** Flow chart of the selection of patients affected by strongyloidiasis analysed in the present study. The population represents a sub-group of patients enrolled in the context of the Strong Treat clinical trial [24]. ^a^Only patients enrolled at the study site IRCCS Sacro Cuore Don Calabria Hospital (Negrar, Verona, Italy) were considered. **Table S1.** Immune factor levels measured in uninfected control subjects (CTRL), infected subjects at baseline (Ss^+^ BT) and 6 months after treatment (6M AT).** Table S2. **Spearman correlation between subjects’ age (*n* = 66) and immune factor concentration at baseline. **Table S3. **ROC analysis (**a**) and marker combination (**b**) for the discrimination of infected and uninfected subjects.** Table S4.** Evaluation of immune factor levels in patients presenting clinical symptoms (*n* = 22) and those without symptoms (*n* = 10) at baseline. **Table S5.** Summary of published studies investigating the host immune response in the human host against *S. stercoralis.*

## Data Availability

The de-identified dataset supporting the conclusions of this article will be made available upon publication in Mendeley Data repository.

## Abbreviations

Ss^+^: Patients diagnosed with strongyloidiasis
BT: Before ivermectin treatment
6M AT: Six months after ivermectin treatment
CTRL: Uninfected controls
Th1: T helper type 1 (immune response)
Th2: T helper type 2 (immune response)
IL: Interleukin
HTLV: Human T cell lymphotropic virus (or human T-cell leukemia virus)
IFAT: Immunofluorescent test
OD: Optical density
CXCL: C-X-C motif chemokine
CCL: C-C motif chemokine
bFGF: Basic fibroblast growth factor
G-CSF: Granulocyte-colony stimulating factor
GM-CSF: Granulocyte-macrophage colony-stimulating factor
IFN-γ: Interferon gamma
IP: Interferon gamma-induced protein
MCP: Monocyte chemoattractant protein
MIP: Macrophage inflammatory proteins
PDGF-BB: Platelet-derived growth factor subunit B
TNF: Tumour necrosis factor
VEGF: Vascular endothelial growth factor
CV: Coefficient of variation
OOR: Out of range
ROC: Receiver operator characteristic
SE: Sensitivity
SP: Specificity
WBC: White blood cell
AUC: Area under the curve
