# Supplementary material for: Systemic profile of immune factors in an elderly Italian population affected by chronic strongyloidiasis
Source: Parasit Vectors. 2020 Oct 15;13:515. doi: 10.1186/s13071-020-04391-w (PMC7559927; doi:10.1186/s13071-020-04391-w)
Supplement: Supplementary file 1 — Additional file 1: Figure S1. Flow chart of the selection of patients affected by strongyloidiasis analysed in the present study. The population represents a sub-group of patients enrolled in the context of the Strong Treat clinical trial [24]. aOnly patients enrolled at the study site IRCCS Sacro Cuore Don Calabria Hospital (Negrar, Verona, Italy) were considered. Table S1. Immune factor levels measured in uninfected control subjects (CTRL), infected subjects at baseline (Ss+ BT) and 6 months after treatment (6M AT). Table S2. Spearman correlation between subjects’ age (n = 66) and immune factor concentration at baseline. Table S3. ROC analysis (a) and marker combination (b) for the discrimination of infected and uninfected subjects. Table S4. Evaluation of immune factor levels in patients presenting clinical symptoms (n = 22) and those without symptoms (n = 10) at baseline. Table S5. Summary of published studies investigating the host immune response in the human host against S. stercoralis. [file 13071_2020_4391_MOESM1_ESM.pdf]

Additional material

Systemic profile of immune factors in an elderly Italian population affected by chronic strongyloidiasis

Natalia Tiberti[1], Dora Buonfrate[1], Carmine Carbone[2], Geny Piro[2], Zeno Bisoffi[1,3], Chiara Piubelli[1]

Figure S1. Flow chart of the selection of patients affected by strongyloidiasis analysed in the present study. The population represents a sub-group of patients enrolled in the context of the Strong Treat clinical trial [24]. [a] Only patients enrolled at the study site IRCCS Sacro Cuore Don Calabria Hospital (Negrar, Verona, Italy) were considered. The flow chart was generated with Lucidchart (Lucid Software Inc.).

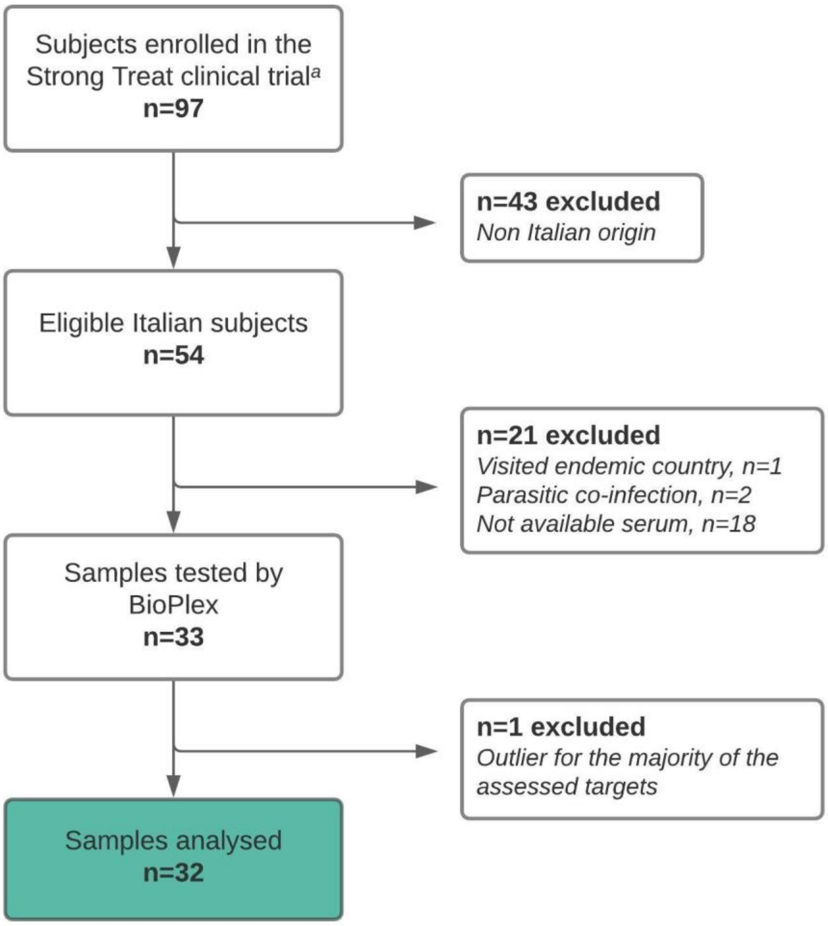

Table S1. Immune factor levels measured in uninfected control subjects (CTRL), infected subjects at baseline (Ss^+ BT) and 6 months after treatment (6M AT).

	CTRL (n=34)	Ss^+ BT (n=32)	Ss^+ 6M AT (n=32)	CTRL vs Ss^+ BT[a]	Ss^+ BT vs 6M AT[b]
**Target [pg/mL]**	**Median (range)**	**Median (range)**	**Median (range)**	**p-value**	**p-value**
Th1 cytokines					
**IL-2**	3.52 (1.22 - 9.42)	3.52 (1.22 - 11.01)	2.82 (1.22 - 5.69)	0.5665	**0.0337***
**IL-12p70**	0.44 (0.44 - 3.50)	0.44 (0.44 - 13.01)	0.44 (0.44 - 3.50)	0.1209	0.05
**TNF**	88.98 (61.90 - 166.09)	79.37 (53.08 - 124.24)	80.47 (57.63 - 126.17)	**0.0382***	0.2267
**IFN-γ**	3.75 (0.63 - 18.00)	3.75 (0.60 - 26.73)	3.08 (0.34 - 13.15)	0.8070	**0.0113***
Th2 cytokines					
**IL-4**	3.09 (1.86 - 7.27)	3.24 (2.06 - 24.17)	3.48 (2.66 - 21.56)	0.4486	0.1010
**IL-6**	2.98 (0.05 - 30.23)	1.49 (0.05 - 64.55)	1.80 (0.05 - 10.25)	0.0750	0.9701
**IL-9**	184.40 (108.45 - 256.59)	161.97 (76.99 - 223.11)	166.89 (125.09 - 225.60)	**0.0224***	0.1499
**IL-13**	3.74 (0.37 - 36.57)	4.12 (0.15 - 58.61)	2.96 (0.37 - 27.04)	0.1970	0.1140
Th17 cytokines					
**IL-17A**	15.72 (10.06 - 39.95)	13.35 (6.92 - 19.49)	14.60 (10.15 - 20.16)	0.2287	0.1493
Chemokines					
**IL-8 (CXCL8)**	17.46 (6.34 - 628.45)	12.95 (4.01 - 51.36)	11.99 (3.16 - 97.05)	**0.0432***	0.7015
**IP-10 (CXCL10)**	1220.25 (363.93 – 12874.76)	977.11 (277.05 – 13009.98)	844.43 (205.21 – 2635.06)	0.1300	0.2242
**CCL2 (MCP-1)**	40.76 (9.75 - 110.76)	36.07 (11.40 - 71.64)	37.74 (17.45 - 84.26)	0.3900	0.4322
**CCL3 (MIP-1α)**	4.22 (1.44 - 34.85)	3.13 (1.52 - 9.86)	3.54 (1.41 - 10.92)	**0.0105***	0.0788
**CCL4 (MIP-1β)**	137.10 (84.36 - 225.08)	124.22 (48.31 - 182.19)	133.44 (98.92 - 172.80)	**0.0239***	**0.0209***
**CCL5 (Rantes)**	16595.39 (3419.38 – 264984.20)	14503.84 (912.68 – 264984.20)	14856.01 (5485.77 – 264984.20)	**0.0435***	**0.0338***
**CCL11 (Eotaxin)**	105.70 (53.21 - 526.20)	102.40 (46.33 - 211.21)	132.17 (77.42 - 448.42)	0.7194	**<0.0001*****
Other cytokines & growth factors					
**IL-1β**	0.53 (0.22 - 14.07)	0.53 (0.13 - 8.25)	0.44 (0.22 - 2.41)	0.6159	0.0869
**IL-1ra**	310.03 (122.05 – 2457.89)	238.09 (126.44 – 3715.87)	208.45 (108.45 - 588.60)	0.0879	**0.0267***
**IL-7**	17.98 (5.46 - 63.70)	17.30 (9.69 - 34.26)	19.20 (8.35 - 47.20)	0.8068	**0.0403***
**bFGF**	44.70 (26.94 - 58.54)	41.35 (18.76 - 52.44)	43.03 (34.38 - 52.44)	**0.0261***	**0.0087****
**G-CSF**	204.08 (70.83 - 894.58)	158.41 (62.39 - 454.48)	160.68 (104.92 - 367.94)	**0.0426***	0.4948
**PDGF-BB**	4602.90 (447.36 – 11526.77)	4279.53 (76.37 – 10748.48)	5290.53 (1628.43 – 8732.89)	0.1698	**0.0053****
**VEGF**	16.14 (16.14 – 1623.77)	61.43 (16.14 - 878.78)	16.14 (16.14 - 281.51)	0.1549	0.0749

BT: before treatment (baseline); 6M AT: 6 months after treatment. [a] Mann-Whitney U test. [b] Wilcoxon signed-rank test

Table S2. Spearman correlation between subjects' age (n=66) and immune factor concertation at baseline.

Target [pg/mL]	Spearman *rho*	95% confidence interval	*P*-value
Th1 cytokines			
**IL-2**	**0.2747**	0.0277 - 0.4901	**0.0256***
**IL-12p70**	**0.3137**	0.0703 - 0.5218	**0.0103***
**TNF**	-0.0039	-0.2525 - 0.2453	0.9755
**IFN-γ**	0.0865	-0.1659 - 0.3284	0.4896
Th2 cytokines			
**IL-4**	0.0929	-0.1597 - 0.3341	0.4582
**IL-6**	0.1467	-0.1061 - 0.3816	0.2399
**IL-9**	-0.0686	-0.3121 - 0.1834	0.5843
**IL-13**	0.0459	-0.2053 - 0.2915	0.7142
Th17 cytokines			
**IL-17A**	0.1494	-0.1033 - 0.3840	0.2311
Chemokines			
**IL-8 (CXCL8)**	0.2413	-0.0080 - 0.4624	0.0509
**IP-10 (CXCL10)**	0.2250	-0.0253 - 0.4488	0.0693
**CCL2 (MCP-1)**	-0.1345	-0.3710 - 0.1184	0.2816
**CCL3 (MIP-1α)**	0.1291	-0.1238 - 0.3662	0.3015
**CCL4 (MIP-1β)**	-0.0995	-0.3400 - 0.1531	0.4265
**CCL5 (Rantes)**	**-0.3775**	-0.5726 - -0.1419	**0.0018****
**CCL11 (Eotaxin)**	0.0272	-0.2232 - 0.2743	0.8281
Other cytokines & growth factors			
**IL-1b**	0.0935	-0.1591 - 0.3346	0.4550
**IL-1ra**	0.1050	-0.1478 - 0.3448	0.4016
**IL-7**	0.0967	-0.1560 - 0.3375	0.4400
**bFGF**	-0.222	-0.4462 - 0.0285	0.0733
**GC-CSF**	0.2054	-0.0458 - 0.4322	0.0980
**PDGF-BB**	-0.2248	-0.4486 - 0.0255	0.0695
**VEGF**	0.1734	-0.0789 - 0.4048	0.1639

Table S3. ROC analysis (A) and marker combination (B) for the discrimination of infected and uninfected subjects.

a. Individual markers

Marker	% AUC (95% CI)	Cut-off	%SP (95% CI)	%SE (95% CI)
**Eosinophilia** (>400 cells/µl)	73 (62.3-83.6)	0.5	64.7 (47.1-79.4)	81.2 (68.8-93.8)
**TNF** [pg/ml]	64.8 (51.6-78.1)	92.39	47.1 (29.4-64.7)	78.1 (62.5-90.6)
**IL-9** [pg/ml]	66.4 (53.1-79.7)	153.445	85.3 (73.5-94.2)	43.8 (25.0-59.4)
**IL-8** [pg/ml]	64.5 (51.0-77.9)	10.545	88.2 (76.5-97.1)	40.6 (25.0-59.4)
**CCL3** [pg/ml]	68.3 (55.3-81.4)	6.83	38.2 (23.5-55.9)	96.9 (90.6-100.0)
**CCL4** [pg/ml]	66.2 (53.0-79.4)	126.29	73.5 (58.8-88.2)	56.2 (40.6-75.0)
**CCL5** [pg/ml]	64.4 (50.9-78.0)	13612.8	82.4 (67.6-94.1)	50 (34.4-65.6)
**bFGF** [pg/ml]	65.9 (52.7-79.1)	50.22	32.4 (17.6-47.1)	96.9 (90.6-100.0)
**G-CSF** [pg/ml]	64.5 (50.9-78.1)	220.185	47.1 (32.4-64.7)	87.5 (75.0-96.9)

b. Marker combination

Marker	Cut-off	% AUC (95% CI)	%SP (95% CI)	%SE (95% CI)	*P*-value[a]
**Eosinophilia** (>400 cells/µl)	>0.5	87.6 (79.2-96.0)	85.3 (73.5-97.1)	87.5 (75.0-96.9)	0.0005
**IL-9** [pg/ml]	<144.205				
**CCL3** [pg/ml]	<6.83				

Panel positive when any 2 markers are positive
AUC= area under the ROC curve, SP= specificity, SE= sensitivity, 95% CI= 95% confidence interval
[a] *De Long's test comparing the AUC of the panel and the one of the best individual marker, i.e., eosinophilia.*

Table S4. Evaluation of immune factor levels in patients presenting clinical symptoms (n=22) and those without symptoms (n=10) at baseline.

	**Absence of symptoms at baseline (n=10)**	**Presence of symptoms at baseline (n=22)**	**Mann-Whitney U test**
**Target [pg/mL]**	**Median (range)**	**Median (range)**	**p-value**
Th1 cytokines			
**IL-2**	4.61 (2.97 - 11.01)	2.82 (1.22 - 5.69)	**0.0009*** **
**IL-12p70**	3.08 (0.44 - 13.01)	0.44 (0.44 - 5.50)	**0.0043** **
**TNF**	91.39 (53.08 - 124.24)	76.60 (54.77 - 112.59)	0.1796
**IFNγ**	4.73 (2.90 - 26.73)	3.75 (0.60 - 24.85)	0.3279
Th2 cytokines			
**IL-4**	4.01 (3.34 - 7.51)	2.85 (2.06 - 24.17)	**0.0005*** **
**IL-6**	2.85 (0.40 - 64.55)	0.80 (0.05 - 23.65)	0.1262
**IL-9**	183.05 (76.99 - 223.11)	157.94 (79.05 - 219.37)	0.3711
**IL-13**	7.09 (2.04 - 58.61)	3.60 (0.15 - 18.48)	0.0642
Th17 cytokines			
**IL-17A**	17.49 (11.22 - 19.49)	12.02 (6.92 - 17.55)	**0.0027** **
Chemokines			
**IL-8 (CXCL8)**	20.04 (10.06 - 33.51)	9.70 (4.01 - 51.36)	**0.0227* **
**IP-10 (CXCL10)**	898.29 (355.02 – 13009.98)	1013.31 (277.05 – 3080.04)	0.8230
**CCL2 (MCP-1)**	42.03 (18.53 - 71.64)	31.66 (11.40 - 69.46)	0.0840
**CCL3 (MIP-1α)**	3.27 (2.09 - 9.86)	3.13 (1.52 - 5.84)	0.3601
**CCL4 (MIP-1β)**	138.52 (48.31 - 182.19)	122.85 (82.12 - 170.80)	0.4895
**CCL5 (Rantes)**	12053.76 (912.68 – 264984.2)	16232.3 (2067.51 – 264984.2)	0.2074
**CCL11 (Eotaxin)**	140.57 (85.45 - 211.21)	95.51 (46.33 - 150.88)	**0.0039** **
Other cytokines & growth factors			
**IL-1β**	0.75 (0.33 - 8.25)	0.45 (0.13 - 5.04)	**0.0396* **
**IL-1ra**	275.67 (143.45 – 3715.87)	223.39 (126.44 – 1828.05)	0.1273
**IL-7**	23.06 (15.82 - 25.56)	15.82 (9.69 - 34.26)	**0.0029* **
**bFGF**	41.35 (18.76 - 47.96)	42.02 (27.98 - 52.44)	0.6246
**G-CSF**	196.49 (144.60 - 454.48)	130.24 (62.39 - 219.38)	**0.0006*** **
**PDGF-BB**	4283.80 (76.37 – 7473.29)	4279.53 (501.53 – 10748.48)	0.4643
**VEGF**	93.29 (16.14 - 878.78)	34.15 (16.14 - 253.79)	0.2067

Clinical symptoms were defined as: skin rush, abdominal pain, pruritus, respiratory symptoms

Table S5. Summary of published studies investigating the host immune response in the human host against *S. stercoralis*. Only studies analysing clinical samples have been considered.

Compared conditions	Study design	IFN-γ	TNF	IL-2	IL-4	IL-5	IL-13	IL-10	Other factors tested	Main findings	Ref.
S. stercoralis infection only											
Ss$^+$ vs. Uninfected controls	Supernatant of isolated CD4$^+$ T cells, unstimulated or antigen-activated	↓	↓	↓	↑	↑	↑		↓ IL-17, IL-22	*S. stercoralis* infection is associated with increased frequencies of CD4$^+$ T cells secreting Th2 cytokines and decreased frequency of CD4$^+$ T cells secreting Th1 and Th17 cytokines. This profile is reverted following anti-helminthic treatment.	[1]
Ss$^+$ after tt vs. *Ss*$^+$ before tt	Supernatant of isolated CD4$^+$ T cells, unstimulated or antigen-activated	↑	↑	↑	↓	↓	↓		↑ IL-22		
Ss$^+$ vs. Uninfected controls	Plasma	↓	↓	—	↑	↑	↑	↑	↓ IL-1b<br>↑ IL-9, IL-27, IL-37, TGFβ	Asymptomatic strongyloidiasis is associated with increased plasma concentration of anti-inflammatory cytokines and decreased concentration of pro-inflammatory cytokines. This profile is reverted following anti-helminthic treatment.	[2]
Ss$^+$ after tt vs. *Ss*$^+$ before tt	Plasma	↑	↑	↑	↓	↓	↓	↓	↑ IL-17A, IL-18, IL-22, Il-23, IL-1b<br>↓ IL-9, IL-27, IL-37, TGFβ		
Ss$^+$ vs. Uninfected controls	Plasma							↑	↑ IFN-λ1, IFN-λ2, IFN-λ3, CXCL10	*S. stercoralis* infection is characterised by elevated systemic and antigen-induced levels of type III IFNs and CXCL10. This profile is reverted following treatment.	[3]
Ss$^+$ after tt vs. *Ss*$^+$ before tt	Plasma							↓	↓ IFN-λ1, IFN-λ2, IFN-λ3, CXCL10		
Ss$^+$ vs. Uninfected controls	Whole blood supernatant, unstimulated or antigen stimulated								↑ IFN-λ1, IFN-λ2, IFN-λ3, CXCL10		

Ss+ vs. Uninfected controls	Plasma			↓	↑				↓ IL-7, IL-15<br>↑ IL-9	Association between γc cytokines and strongyloidiasis.	[4]
Ss+ after tt vs. *Ss*+ before tt	Plasma			↑	↓				↑ IL-7, IL-15<br>↓ IL-9		
Ss+ vs. Uninfected controls	Plasma								↑ IL-6, IL-8, MCP-1, MIP-1β<br>↑ acute phase proteins<br>↑ microbial translocation markers	*S. stercoralis* infection might be associated with microbial translocation.	[5]
S. stercoralis infection + co-morbidities											
LTB+/*Ss*+ vs. LTB+	Whole blood supernatant, stimulated with TB antigens or mitogen								↓ CCL1, CCL2, CCL4, CCL11, CXCL9, CXCL10, CXCL11	*S. stercoralis* significantly modulates the expression of chemokines in response to TB antigens.	[6]
LTB+/*Ss*+ after tt vs LTB+/*Ss*+ before tt	Whole blood supernatant								↑ CCL1, CCL2, CCL4, CCL11, CXCL11		
ATB+/*Ss*+ vs. ATB+	Plasma	↓	↓	↓	—	—	—	↑	↓ IL-17A<br>↑ IL-22, TGFβ	*S. stercoralis* shows immunomodulatory effects on the systemic cytokine levels in response to active and latent tuberculosis.	[7]
LTB+/*Ss*+ vs. LTB+	Plasma	↓	↓	↓	↑	↑	↑	↑	↓ IL-17A, IL-17F<br>↑ TGFβ		
T2DM+/*Ss*+ vs. T2DM+	Plasma								↓ IL-1α, IL-1β, IL-1ra, IL-6, IL-12, IL-18, IL-23, IL-27, G-CSF, GM-CSF, CCL1, CCL2, CCL3, CCL11, CXCL1, CXCL2, CXCL8, CXCL9, CXCL10,CXCL11	*S. stercoralis* infection induces a shift towards a reduced pro-inflammatory status in T2DM patients. The co-morbidity with *S. stercoralis* might provide protection from T2DM associated pathology.	[8]

T2DM^{+}/*Ss*$^{+}$ after tt T2DM^{+}/*Ss*$^{+}$ before tt	Plasma								↑ IL-1α, IL-1β, IL-6, IL-12, IL-18, IL-23, IL-27, G-CSF, GM-CSF, CCL1, CCL2, CCL3, CXCL1, CXCL2, CXCL8, CXCL9, CXCL10, CXCL11↓ IL-1ra		
T2DM^{+}/*Ss*$^{+}$ vs. T2DM^{+}	Plasma	↓	↓	↓	↑	↑	↑	—	↓ IL-17A, IL-17F, IL-22 ↑ TGFβ	The co-infection with *S. stercoralis* modulates the host immune response to T2DM, suggesting that *S. stercoralis* may provide a degree of protection against the severity of T2DM.	[9]
Ss^{+} vs. HTLV-1^{+}/*Ss*$^{+}$	Isolated PBMCs stimulated with *S. stercoralis* antigens	↓				↑	—	↓	none	The co-infection with HTLV-1 induces a shift towards a Th1 response in patients infected with *S. stercoralis.*	[10]
Ss^{+} vs. HTLV-1^{+}/*Ss*$^{+}$	Isolated lymphocytes	↓	↓			—		—	↑ sIL-2R	*S. stercoralis* is proposed to modulate the host immune response to HTLV-1 through a decreased Th1 immune response, which is restored after successful anti-helminthic treatment.	[11]
HTLV-1$^{+/}$helminths^{+} vs. HTLV-1^{+}	Isolated CD8^{+} or CD4^{+} T cells	↓				↑		↑	none	Helminthic infections (*S. stercoralis* or *S. mansoni*) decrease the production of IFNγ and the overall frequency of CD8+ and CD4+ expressing IFNγ in HTLV-1 carriers. The frequency of IL-10 expressing cells is higher in co-infected patients.	[12]
Ss^{+} vs. HTLV-1^{+}/*Ss*$^{+}$	Isolated PBMCs unstimulated or mitogen stimulated	↓			↑				↑ IgE	In patients affected by *S. stercoralis* there is no spontaneous release of IFNγ, but a substantial IL-4 production upon stimulation. Increased IFNγ in co-infected subjects might inhibit IL-4 and consequently IgE production, thus	[13]

										modulating the immune response to *S. stercoralis.*	
Ss+<br>vs.<br>HTLV-1+/*Ss*+	Isolated PBMCs unstimulated or antigen stimulated					↑				Patients with co-infection have increased Treg and decreased eosinophils and IL-5 production in response to antigen stimulation compared to patients infected with *S. stercoralis* only.	[14]
Present study											
Ss+<br>vs.<br>Uninfected controls	Serum	—	↓	—	—		—		↓ IL-9, IL-8, CCL3, CCL4, CCL5, bFGF, PDGF-BB	Elderly patients infected with *S. stercoralis* for decades in the absence of re-infection display an overall dampened immune response. In these patients, chemokines are decreased at baseline compared to uninfected controls and reverted after treatment. The immune response against auto-infective strongyloidiasis may differ from the one generated in the presence of re-infection.	
Ss+ after tt<br>vs.<br>*Ss*+ before tt	Serum	↓	—	↓	—		—		↑ CCL4, CCL5, CCL11, bFGF, IL-7, PDGF-BB<br>↓ IL-1ra		

The most commonly investigated Th1 (IFNγ, TNF, IL-2) and Th2 (IL-4, IL-5, IL-13) cytokines have been represented in individual columns to facilitate data comparison. Other factors tested in the study have been reported in an additional column. The arrows indicate whether a cytokines is increased or reduced in concentration for the indicated comparison.

References:

1. Anuradha R, Munisankar S, Dolla C, Kumaran P, Nutman TB, Babu S. Parasite antigen-specific regulation of th1, th2, and th17 responses in strongyloides stercoralis infection. J Immunol 2015; 195(5):2241-2250.
2. Anuradha R, Munisankar S, Bhootra Y, Jagannathan J, Dolla C, Kumaran P, et al. Systemic cytokine profiles in strongyloides stercoralis infection and alterations following treatment. Infect Immun 2016; 84(2):425-431.
3. Rajamanickam A, Munisankar S, Bhootra Y, Dolla C, Nutman TB, Babu S. Elevated systemic and parasite-antigen stimulated levels of type iii ifns in a chronic helminth infection and reversal following anthelmintic treatment. Front Immunol 2018; 9:2353.
4. Rajamanickam A, Munisankar S, Bhootra Y, Dolla CK, Thiruvengadam K, Nutman TB, et al. Altered levels of memory t cell subsets and common gammac cytokines in strongyloides stercoralis infection and partial reversal following anthelmintic treatment. PLoS Negl Trop Dis 2018; 12(5):e0006481.
5. Rajamanickam A, Munisankar S, Bhootra Y, Dolla C, Nutman TB, Babu S. Microbial translocation associated with an acute-phase response and elevations in mmp-1, ho-1, and proinflammatory cytokines in strongyloides stercoralis infection. Infect Immun 2017; 85(1).

6. Rajamanickam A, Munisankar S, Bhootra Y, Dolla CK, Nutman TB, Babu S. Coexistent helminth infection-mediated modulation of chemokine responses in latent tuberculosis. J Immunol 2019; 202(5):1494-1500.
7. George PJ, Pavan Kumar N, Jaganathan J, Dolla C, Kumaran P, Nair D, et al. Modulation of pro- and anti-inflammatory cytokines in active and latent tuberculosis by coexistent strongyloides stercoralis infection. Tuberculosis (Edinb) 2015; 95(6):822-828.
8. Rajamanickam A, Munisankar S, Dolla C, Menon PA, Thiruvengadam K, Nutman TB, et al. Helminth infection modulates systemic pro-inflammatory cytokines and chemokines implicated in type 2 diabetes mellitus pathogenesis. PLoS Negl Trop Dis 2020; 14(3):e0008101.
9. Rajamanickam A, Munisankar S, Bhootra Y, Dolla C, Thiruvengadam K, Nutman TB, et al. Metabolic consequences of concomitant strongyloides stercoralis infection in patients with type 2 diabetes mellitus. Clin Infect Dis 2019; 69(4):697-704.
10. Porto AF, Neva FA, Bittencourt H, Lisboa W, Thompson R, Alcantara L, et al. Htlv-1 decreases th2 type of immune response in patients with strongyloidiasis. Parasite Immunol 2001; 23(9):503-507.
11. Salles F, Bacellar A, Amorim M, Orge G, Sundberg M, Lima M, et al. Treatment of strongyloidiasis in htlv-1 and strongyloides stercoralis coinfected patients is associated with increased tnfalpha and decreased soluble il2 receptor levels. Trans R Soc Trop Med Hyg 2013; 107(8):526-529.
12. Porto AF, Santos SB, Muniz AL, Basilio V, Rodrigues W, Jr., Neva FA, et al. Helminthic infection down-regulates type 1 immune responses in human t cell lymphotropic virus type 1 (htlv-1) carriers and is more prevalent in htlv-1 carriers than in patients with htlv-1-associated myelopathy/tropical spastic paraparesis. J Infect Dis 2005; 191(4):612-618.
13. Neva FA, Filho JO, Gam AA, Thompson R, Freitas V, Melo A, et al. Interferon-gamma and interleukin-4 responses in relation to serum ige levels in persons infected with human t lymphotropic virus type i and strongyloides stercoralis. J Infect Dis 1998; 178(6):1856-1859.
14. Montes M, Sanchez C, Verdonck K, Lake JE, Gonzalez E, Lopez G, et al. Regulatory t cell expansion in htlv-1 and strongyloidiasis co-infection is associated with reduced il-5 responses to strongyloides stercoralis antigen. PLoS Negl Trop Dis 2009; 3(6):e456.
